# Polychromatic immunophenotypic characterization of T cell profiles among HIV-infected patients experiencing immune reconstitution inflammatory syndrome (IRIS)

**DOI:** 10.1186/1742-6405-6-16

**Published:** 2009-07-16

**Authors:** David M Murdoch, Melinda S Suchard, Willem DF Venter, Patrick Mhlangu, Janet S Ottinger, Charles Feldman, Annelies Van Rie, Deborah K Glencross, Wendy S Stevens, Kent J Weinhold

**Affiliations:** 1 Division of Pulmonary and Critical Care Medicine, Duke University Medical Center, Durham, North Carolina, USA; 2 Department of Molecular Medicine and Haematology, University of the Witwatersrand and National Health Laboratory Services, Johannesburg, South Africa; 3 Reproductive Health & HIV Research Unit, University of the Witwatersrand, Johannesburg, South Africa; 4 Contract Laboratory Services, Johannesburg, South Africa; 5 Department of Surgery and Immunology and the Duke University Center for AIDS Research (CFAR), Duke University, Durham, North Carolina, USA; 6 Division of Pulmonology, Department of Medicine, Johannesburg Hospital and University of the Witwatersrand, Johannesburg, South Africa; 7 Department of Epidemiology, The University of North Carolina at Chapel Hill, Chapel Hill, NC, USA

## Abstract

**Objective:**

To immunophenotype CD4^+ ^and CD8^+ ^T cell sub-populations in HIV-associated immune reconstitution inflammatory syndrome (IRIS).

**Design:**

Nested case-control immunological study.

**Methods:**

ART-naïve HIV-infected patients were prospectively observed for IRIS during the first 6 months of ART. Twenty-two IRIS cases and 22 ART-duration matched controls were sampled for T cell immunophenotyping.

**Results:**

IRIS cases demonstrated significantly lower CD4 cell counts compared to controls (baseline: 79 versus 142, p = 0.02; enrollment: 183 versus 263, p = 0.05, respectively) with no differences in HIV RNA levels. Within CD4^+^T cells, cases exhibited more of an effector memory phenotype compared to controls (40.8 versus 27.0%, p = 0.20), while controls trended towards a central memory phenotype (43.8 versus 30.8%, p = 0.07). Within CD8^+ ^T cells, controls exhibited more central memory (13.9 versus 7.81%, p = 0.01, respectively) and effector (13.2 versus 8.8%, p = 0.04, respectively) phenotypes compared to cases, whereas cases demonstrated more terminal effectors than controls (28.8 versus 15.1%, p = 0.05). Cases demonstrated increased activation of CD8^+ ^T cell effector memory, terminal effector, and effector subsets than controls (p = 0.04, 0.02, and 0.02, respectively).

**Conclusion:**

CD4^+ ^and CD8^+ ^T cell subset maturational phenotypes were heterogeneous among IRIS cases and controls. However, IRIS cases demonstrated significant increases in activation of CD8^+ ^T cell effector subpopulations.

## Background

The immune reconstitution inflammatory syndrome (IRIS) or immune reconstitution disease (IRD) is a clinical deterioration occurring in the first few weeks following the introduction of antiretroviral therapy (ART) [1,2]. This clinical deterioration is due to immune restoration and a subsequent inflammatory response to a wide variety of intact subclinical pathogens and/or residual antigen[3]. Incidence estimates of the syndrome vary depending on the population studied and case definitions used, but suggest IRIS affects a significant proportion (10–32%) of patients initiating ART [2,4-7].

It is speculated that IRIS results from the restoration of immunity to pathogen-specific antigens present at the time of ART initiation. Following ART, a quantitative increase in peripheral T cells occurs which partially restores activity to recall antigens as a result of suppression of HIV viral replication in lymphoid tissue and reductions in immune activation [8,9]. Furthermore, restoration of *in vitro *responses to common infectious antigens and improvement in delayed type hypersensitivity has been significantly associated with increases in memory CD4 T cells following ART [10].

Although the immunopathogenesis of the syndrome remains largely unknown, these observations have resulted in the hypothesis that IRIS results, at least in part, from the restoration of immunity to pathogen specific antigens present at the time or ART initiation and from redistribution of antigen-specific memory T cells with tissue-specific localized inflammation [3,11]. This theory is further supported by the observation of an exuberant pro-inflammatory Th1 response to mycobacterial antigens in IRIS patients independent of the degree of absolute CD4 cell recovery [12]. To date, studies have suggested a predominance of memory phenotype CD4^+ ^cells in localized inflammatory tissue [11]. However, these results are difficult to interpret given the lymphocyte redistribution following ART in all HIV-infected subjects and the lack of non-IRIS patients for comparison.

In order to test the hypothesis that IRIS results, in part, from the peripheral redistribution of a greater number of T cells with a predominant memory phenotype, we performed immunophenotyping to characterize T cell populations in IRIS and non-IRIS subjects. We also examined differences in immune activation between groups in an attempt to understand the immunopathogenesis of the syndrome.

## Methods

### Study Population

Participants consisted of confirmed IRIS cases and controls selected from an HIV-infected ART-naïve adult (>18 years) cohort who participated in a prospective surveillance study to examine the incidence of IRIS in Sub-Saharan Africa between January 6, 2006 and July 7, 2007 [5]. ART initiation criteria were in accordance with the South Africa National ART Program and included a CD4 count ≤200 cells/mm^3 ^or WHO stage IV AIDS-defining illness irrespective of CD4 count. Patients received scheduled clinical assessments, including at least one pre-treatment assessment, one treatment commencement assessment, and regularly scheduled assessments at weeks 2, 4, 8 and every 3 months thereafter [13]. Enrollment into the nested case-control study required participation in the prospective surveillance cohort, a pre-treatment CD4 count and HIV RNA level, and willingness to provide written informed consent for an additional blood draw and sample storage.

In general, IRIS is clinically defined as a paradoxical clinical worsening due to a subclinical opportunistic pathogen ("unmasking" IRIS) or previously known treated (completed or ongoing) opportunistic pathogen ("paradoxical" IRIS) in the setting of an adequate response to ART [14-16]. For the "unmasking" form of IRIS, a new localized infection was required from a focal inflammatory process (suppurative lymph node, pulmonary infiltrate, positive CSF culture, etc.) in a patient who, prior to ART, exhibited no signs or symptoms of disease and in whom adequate OI screening and clinical assessment had been performed (i.e. negative pre-ART sputum AFBs in the case of "unmasking" pulmonary TB). For organisms for which cultures or diagnostic studies were available (i.e. TB, Cryptococcus) demonstration of the organism or a pathological process characteristic of the organism (i.e. caseous necrosis, granulomatous inflammation) were required. For the "paradoxical" form of IRIS, a patient required the diagnosis and treatment initiation of an OI prior to ART initiation with a positive clinical response. Following ART, the patient experienced a new inflammatory process (worsening lymphadenopathy or suppuration, expansion of Kaposi's lesions, recurrence of meningeal signs and symptoms) at the original or new site of infection accompanied by systemic symptoms (fever, loss of weight, elevated white blood cell count). For all cases, no other identifiable pathogen could be present after thorough diagnostic evaluation.

For this immunological analysis, only *confirmed *IRIS cases were examined. These consisted of subjects who: 1) exhibited symptoms consistent with an infectious or inflammatory condition while on ART which could not be explained by the expected clinical course of a previously recognized infectious agent or by side effects of therapy, *and *2) whose treatment led to a ≥1 log_10 _drop in HIV RNA at time of IRIS diagnosis, and 3) whose treatment resulted in adequate immune reconstitution 6 months post ART, defined as a ≥1 log_10 _drop in HIV RNA or a CD4 count equal to or above the pre-treatment baseline value. Peripheral blood sampling was performed at the time of IRIS diagnosis for IRIS cases and at study enrollment for matched controls. Eligible control subjects were matched on ART duration within ± 2 weeks in a 1:1 ratio. The study protocol was reviewed and approved by all participating institutional review boards.

### CD4 cell count and HIV RNA level measurements

Longitudinal CD4 and CD8 cell counts were measured as previously described [17]. Plasma samples were assayed for HIV-1 RNA levels using the Amplicor polymerase-chain reaction test (Roche Diagnostics, Switzerland).

### Cell isolation, storage, and thawing

Peripheral blood mononuclear cells (PBMCs) were isolated from sodium heparin anticoagulated human blood by density centrifugation (Histopaque, Sigma, Germany). PBMCs were harvested, washed using Hanks Balanced Salt Solution (H9394, Sigma, Germany), and centrifuged at 400 g for 10 minutes. PBMCs were then resuspended in Hanks Balanced Salt Solution and counted using an automated cell counter (Guava Technologies, USA). After counting, 10 million PBMCs/ml were cryopreserved using 1 ml of 10% Dimethyl Sulfoxide (Sigma, Germany) and 90% Fecal Calf Serum (Sigma, Germany) cryopreservation solution. Samples were rate-controlled frozen to -80°C, stored overnight, and then transferred into liquid nitrogen at -180°C until analysis.

Culture medium (R10) was prepared by adding HEPES buffer (1 M), penicillin-streptomycin (1%), sodium pyruvate (100 mM), L-glutamine (200 mM) and heat-inactivated fetal bovine serum (10%) (Sigma, USA) to RPMI 1640 medium (Sigma, Germany). PBMC vials were thawed in a 37°C water bath and 1 ml of R10 with 50 U/ml benzonase (Novagen, Denmark) was added, after which cells were resuspended to 8 ml using R10. Cells were centrifuged for 10 minutes at 250 g, resuspended and washed again using R10. Cells were then counted (Guava Technologies, USA) and rested overnight in a 37°C 5% CO_2 _atmosphere.

### Cell surface staining and measurements

Following overnight rest, 1.0–2.0 × 10^6 ^viable PBMC were stained as previously described[18] using the following optimally titrated monoclonal antibody conjugates in a single tube 9-color panel: HLA-DR APC-Cy7 (L243); CD38 PE Quantibrite (HB7); CD8 Alexa700 (RPA-T8); CD27 APC (L128); CD57 FITC (HNK-1); CD3 AmCyan (SK7); CD4 PerCP Cy5.5 (SK3) (BD Biosciences, CA, USA); CD45RO ECD (UCHL1, Beckman Coulter, France) and vAmine (LIVE/DEAD^® ^Fixable Violet Dead Cell Stain, Invitrogen, USA). An additional 9-color tube consisted of isotype controls for HLA-DR and CD38 using mouse IgG1 APC-Cy7 isotype (X40) and mouse IgG1 PE isotype (X40) (BD Biosciences, USA).

All data were immediately acquired using an LSRII flow cytometer (BD Biosciences, USA) within 4 hours of staining. Between 500,000 and 2 million events were acquired per sample. Compensation was performed daily digitally using FACSDiva software (version 5.0, BD Biosciences, USA) by staining compensation beads (anti-mouse Igκ, BD Biosciences, USA) with the antibodies described with the exception of vAmine which was substituted with CD14 Pacific Blue (BD Biosciences, USA). PMT voltages were checked daily through gating of peak 8 beads (Spherotech, Il, USA). CD38 PE quantitation was performed using CD38 PE Quantibrite and Quantibrite PE Beads (BD Biosciences, USA) according to manufacturer recommended procedures [19].

**Figure 1:**
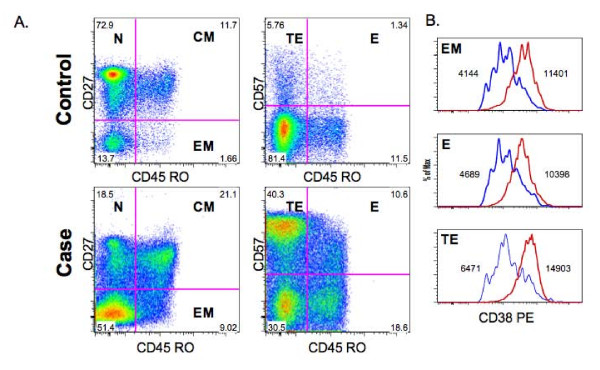
**T cell population maturation and activation profiles for an IRIS case and control subject**. (A). The IRIS case demonstrates greater proportions of CM, TE, and E CD8^+ ^T cell maturational phenotypes compared to the control, which manifests a predominate N and CM phenotype. (B). Differences in CD38 PE expression (activation) within EM, TE, and E subsets between an IRIS case (red line) and respective control (blue line). N: Naïve; CM: Central Memory; EM: Effector Memory; TE: Terminal Effector; E: Effector.

List mode data were analyzed with FlowJo v8.5 (Treestar). Initial singlet gating used a forward scatter width (FSC-W) versus height (FSC-H) plot. Events were then gated through a CD3^+ ^versus vAmine to identify a minimum of 10,000 viable CD3^+ ^lymphocytes and sequentially gated on CD4^+ ^and CD8^+ ^populations. Maturational subsets were defined for CD4^+ ^and CD8^+ ^populations as follows: naïve (N) (CD45RO^-^CD27^+^), central memory (CM) (CD45RO^+^CD27^+^), effector memory (EM) (Effector Memory; CD45RO^+^CD27^-^), E (Effector; CD45RO^+^CD57^+^), & TE (Terminal Effector; CD45RO^-^CD57^+^) [20,21]. Focusing on cells co-expressing CD38 and HLA-DR antigens, cellular activation was measured quantitatively by the number of CD38 PE antibodies bound per cell (ABC), which reflects the density of CD38 antigen on the surface of activated T cells [19]. Cellular activation was measured for each respective CD4^+ ^& CD8^+ ^maturational subset. Figure 1 is an example of gating methods employed for all samples.

### Statistical analyses

Group comparison of baseline clinical and immunological data were performed using the Wilcoxon rank sum and Fisher's exact tests (Stata v10, College Station, USA), where appropriate, with an *a priori *definition of <0.05 considered significant. Group comparisons (IRIS vs. non-IRIS controls) of CD4^+ ^and CD8^+ ^T population subset percentages were performed using the Wilcoxon-Mann-Whitney test. The activation markers CD38 and HLA-DR were gated from CD4^+ ^and CD8^+ ^cells their subsets in which quadrant gates, using an isotype control, were used to define positive and negative populations. For the measurement of CD38 PE ABCs, a minimum of 50 CD38^+ ^HLA-DR^+ ^events were required within each T cell population maturational subset for statistical analysis.

## Results

### Cohort characteristics and clinical outcomes

As reported previously [5], in brief, among the 423 observational cohort patients enrolled, 44 (10.4%) developed IRIS. Clinical manifestations included TB (18/44, 41%), cryptococcal meningitis (3/44, 6.8%), herpes simplex infection (4/44, 9.1%), varicella zoster infection (6/44, 13.6%), molluscum contagiosum (3/44, 6.8%), and Kaposi's sarcoma (2/44, 4.5%), and infectious dermatological manifestations (8/44, 18.2%). With the exception of cases being younger than controls (31 versus 38 years, respectively, p = 0.02), baseline demographics and clinical history were similar between groups (Table 1). The majority (39/44, 89%) were initiated on stavudine/lamivudine/efavirenz.

**Table 1:** Demographic and longitudinal immunologic data for IRIS cases and matched controls

Characteristic*	Controls (n = 22)	Cases (n = 22)	p-value^±^
Black race	22 (100)	22 (100)	0.99
Age (years), median (IQR)	38 (34, 47)	31 (29, 39)	0.02
Female sex	16 (73)	15 (68)	0.99
Duration since HIV diagnosis (months), median (IQR)	5.1 (2.7, 21.2)	2.7 (1.2, 26.8)	0.13
			
No. previous OI/HIV-related illnesses^‡^			
0	4 (18)	8 (36)	0.23
1	10 (45)	8 (36)	
2	5 (23)	6 (28)	
3	3 (14)	0(0)	
			
TB status at ART initiation			
No history of TB	10 (46)	15 (68)	0.26
Therapy completed before ART initiation	6 (27)	2 (9)	
Therapy ongoing at ART initiation	6 (27)	5 (23)	
			
Baseline immunovirologic data			
CD4 cell count	79 (17, 140)	142 (67, 198)	0.02
CD4 cell percentage	7.0 (3.4, 9.0)	7.2 (4.6, 13.6)	0.51
HIV RNA, log_10 _copies/ml	5.4 (4.6, 5.9)	5.4 (4.5, 5.9)	0.77
			
Immunovirologic data at IRIS diagnosis/control enrollment			
CD4 cell count, cells/mm^3§^	183 (66, 252)	263 (152, 337)	0.05
CD4 cell percentage	13.5 (10.4, 20)	14 (10.2, 20.4)	0.96
CD8 cell count, cells/mm^3^	578 (239, 1006)	875 (623, 1487)	0.02
CD8 cell percentage	55 (49, 57)	57 (50, 62)	0.15
CD4:CD8 cell ratio	0.28 (0.19, 0.39)	0.32 (0.14, 0.34)	0.64
HIV RNA, log_10 _copies/ml	2.6 (2.6, 2.7)	2.6 (2.6, 2.6)	0.42
			
Immunovirologic data at 6 months			
CD4 cell count, cells/mm^3§^	161 (99, 294)	277 (158, 372)	0.10
CD4 cell percentage	13.9 (9.8, 20.7)	13.7 (10.2, 21.0)	0.96
HIV RNA, log_10 _copies/ml	2.6 (1.6, 2.6)	2.3 (1.6, 2.6)	0.26

*Data are no. (%) of patients or medians (IQR), unless otherwise indicated. ^± ^p-values for comparison of case/control groups using Wilcoxon rank sum and Fisher's exact tests, where appropriate; <0.05 considered significant.

^‡^As listed by the WHO (Reference number: WHO/HIV/2005.02). ^§^Missing value for 1 IRIS case.

### Immunovirologic outcomes

Of the 44 cases, 22 IRIS cases and 22 matched controls met enrollment criteria for the immunological nested case-control study. Reasons for failure to enroll in the immunological study were prisoner status (n = 2), patient refusal (n = 3), missing baseline or follow-up CD4 count or viral load (n = 4), and failure to enroll patient within two weeks of IRIS identification (n = 13). The median interval of time between ART initiation and the development of IRIS was 38 days (Interquartile range (IQR): 24, 56), with immunological sampling occurring one week later after IRIS diagnosis and clinical evaluations were complete (45 days, IQR: 27–59). Cases demonstrated significantly lower baseline CD4 cell counts compared to controls (79 versus 142 cells/mm^3^, respectively, p = 0.02) with similar baseline HIV RNA levels (Table 1). This observation persisted at IRIS diagnosis and control enrollment (183 versus 263 cells/mm^3^, respectively, p = 0.05) with a continued, but nonsignificant trend by after 6 months of ART (161 versus 277, respectively, p = 0.10). Absolute and percentage changes in CD4 cell count at IRIS diagnosis (for cases) or control enrollment (for matched controls) and at 6 months follow-up were not significant between cases or controls (data not shown). At IRIS diagnosis or control enrollment, controls demonstrated higher absolute CD8 cell counts than cases (875 versus 578 cells/mm^3^, p = 0.02), but no difference in CD8 cell percentage (57 versus 55%, p = 0.15) or CD4:CD8 cell ratio (0.32 versus 0.28, p = 0.64). Response to ART was similar between groups, with no differences in HIV RNA levels at enrollment or at 6 months.

### CD4 and CD8 T cell subset phenotyping

Of the 44 immunologic samples, 3 cases and their respective controls were excluded from flow cytometric analyses secondary to poor cell viabilities and insufficient cell data. Within CD4^+ ^T cells, no significant differences in subset percentages between cases and controls were observed (Table 2). However, both cases and controls demonstrated a predominant CD4^+ ^memory phenotype. Cases trended towards more of an effector memory (EM) phenotype compared to controls (40.8 versus 27.0%, p = 0.20), while controls demonstrated a trend towards an increased central memory (CM) subset (43.8 versus 30.8, p = 0.07).

**Table 2:** Summary of CD4 and CD8 T lymphocyte subset percentages for 19 IRIS cases and 19 matched controls at the time of diagnosis (IRIS cases) or enrollment (controls matched on ART duration)

**T cell subset**	**Controls (n = 19)**	**Cases (n = 19)**	p-value*
**CD4 T lymphocytes**^±^			
CD4+	21.8 (17.2 – 32.5)	21.0 (14.4 – 25.5)	0.38
CD27+ CD45RO- (N)	10.5 (8.44 – 25.3)	15.3 (3.07–25.8)	0.90
CD27+ CD45RO+ (CM)	43.8 (31.3–55.7)	30.8 (13.9 – 50.0)	0.07
CD27- CD45RO+ (EM)	27 (13.2 – 42.8)	40.8 (23.9 – 58.9)	0.20
CD57+ CD45RO- (TE)	0.37 (0.1 – 1.4)	0.38 (0.18 – 2.76)	0.58
CD57+ CD45RO+ (E)	3.99 (1.94 – 10.7)	6.03 (2.63 – 22)	0.17

**CD8 T lymphocytes**^**±**^			
CD8+	70.2 (60.1 – 77)	68.3 (60.5 – 77.4)	0.90
CD27+ CD45RO- (N)	13.3 (9.9 – 17.9)	18.5 (10.3 – 26.6)	0.23
CD27+ CD45RO+ (CM)	13.9 (10.4 – 22.6)	7.81 (3.46 – 12.7)	0.01
CD27- CD45RO+ (EM)	29 (22.7 – 36.1)	20.2 (9.0 – 31.7)	0.08
CD57+ CD45RO- (TE)	15.1 (12.1 – 30.8)	28.8 (18.0 – 42.0)	0.05
CD57+ CD45RO+ (E)	13.2 (7.6 – 24.5)	8.8 (5.5 – 10.7)	0.04

All values determined by manual gating as detailed in methods. All values expressed as median percentage (Interquartile Range) unless otherwise noted. * p-values for comparison of case/control groups using Wilcoxon rank sum test; <0.05 considered significant. ^±^N: Naïve; CM: Central Memory; EM: Effector Memory; TE: Terminal Effector; E: Effector

Within CD8^+ ^T cells, the overall percentage of CD8^+ ^T cells was similar between groups, but subset maturational profiles were heterogeneous. Controls again exhibited significantly higher percentages of central memory (CM) compared to cases (13.9 versus 7.81%, p = 0.01) and exhibited more of an effector (E) phenotype compared to cases (13.2 versus 8.8%, p = 0.04). On the other hand, cases demonstrated an increase in terminal effectors (TE) compared to controls (28.8 versus 15.1%, p = 0.05).

### CD4 and CD8 T cell activation profiles

**Figure 2:**
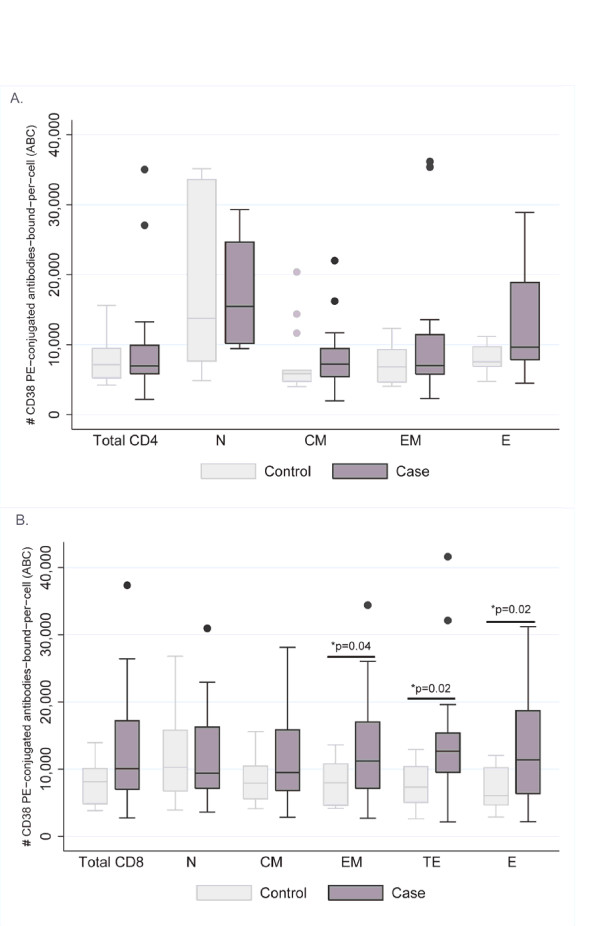
**(A) CD4 T cell CD38 activation patterns between IRIS cases and matched controls**. Insufficient number of events for analysis of TE subset. (B) CD8 T cell CD38 activation patterns between IRIS cases and matched controls. *p-values for comparison of case/control groups using Wilcoxon rank sum test; <0.05 considered significant; ^±^N: Naïve; CM: Central Memory; EM: Effector Memory; TE: Terminal Effector; E: Effector.

T cell activation, as measured by the number of CD38 PE ABCs on respective CD38^+ ^HLA-DR^+ ^T cell subsets, demonstrated significant differences between IRIS cases and matched controls (Figure 2). Within CD8^+ ^subsets, but not within CD4 subsets, cases tended to have more activated CD8^+ ^T cells, demonstrating significantly higher levels of activated effector memory (CD27^-^CD45RO^+^), terminal effectors (CD57^+^CD45RO^-^), and effector (CD57^+^CD45RO^+^) subsets (p = 0.04, 0.02, and 0.02, respectively) and tended to have higher activation levels of central memory subsets (CD27^+^CD45RO^+^) (p = 0.10).

## Discussion

In one of the first attempts to prospectively examine the immunopathogenesis of IRIS, we observed phenotypic heterogeneity across maturational subsets of CD4^+ ^and CD8^+ ^T cells. Interestingly, the surface density of CD38 antigen, a measure of cellular activation previously associated with HIV pathogenesis, was significantly elevated among IRIS subjects. Specifically, IRIS subjects were characterized by higher activation levels of all CD8^+ ^effector subsets (effector memory, terminal effector, and effector).

CD38 and HLA-DR cells surface markers are up regulated upon antigenic stimulation and are used as markers of T cell activation. Varying fluorescence intensity of CD38 expression has been documented in HIV-infected patients prior to commencing therapy [22]. Additionally, CD38 antigen expression levels are correlated with HIV RNA levels and beneficial responses to ART or relapses in therapy [18,22-24]. Increased expression of HLA-DR and CD38 on CD4^+ ^T cells has also been demonstrated in coinfected HIV-infected patients compared to uninfected patients [25]. Although our findings favored activation within CD8^+ ^T cells, these investigators noted similar trends in the CD4^+ ^population. Together with the finding of increased expression of HLA-DR on CD4^+ ^T cells in patients experiencing TB-IRIS [12], our data suggest that T cell activation markers may be indicative of an underlying opportunistic infection and may prove a useful biomarker for identifying patients with activated immune systems and who are at risk for IRIS. Our observation of increases in T cell activation markers in IRIS cases despite similar HIV RNA levels between cases and controls may suggest that immune activation is driven by other factors such as coinfection in addition to HIV RNA levels. How coinfections act synergistically with HIV to drive immune activation and whether T cell activation markers are elevated at baseline in IRIS patients versus non-IRIS patients remain to be fully elucidated.

Increases in activation markers among effector CD8 T cell populations may provide insight into the immunopathogenesis of IRIS. Effector CD8 T cells exhibit specialized functions such as cytotoxicity, antiviral cytokine production, telomerase activity [26,27] and production of cytokines such as IL-2, IFN-γ, TNF-α, perforin, and granzymes A/B/C/K [28,29]. Of these, IFN-γ is known to be increased in response to mycobacterial antigens in patients experiencing TB-IRIS [12,30]. Effector memory cells localize predominately to inflamed non-lymphoid and peripheral tissues where they exhibit immediate effector functions, while central memory T cells express CD62L and CCR7 and localize to lymphoid tissues [31-33]. Because of their peripheral distribution and immediate effector function, effector CD8 T cells may mediate immediate but unsustained responses to peripheral antigens [34,35]. Given that IRIS usually occurs within the first few weeks after ART initiation in patients with lower CD4 cell counts and often involves peripheral tissues such as the lung, a very early and unregulated effector memory response may play a pathogenic role in the syndrome.

The predominance of a CD4^+ ^memory cell phenotype in both cases and controls is in agreement with observations of phenotypic profiles following initiation of ART [8,9]. Although it has been hypothesized that the immunopathogenesis of IRIS may lie within the CD4^+ ^memory cell population [11], we observed no significant phenotypic differences in the percentage of memory cell phenotypes or their activation markers. This may have been due to our limited sample size, peripheral blood compartment sampling, and selection of HIV-infected patients with CD4 cells counts <200 cells/mm^3^. Alternatively, this observation supports the increasing opinion that the manifestations of IRIS due to a variety of pathogens possess distinct immunopathological etiologies. Examples include the role of cytotoxic CD8 T cells in cases of varicella zoster virus IRIS and delayed-type hypersensitivity (DTH) reactions in cases of mycobacterial IRIS [36,37]

The present study had a number of limitations. First, the immunological phenotypes reported here span patients presenting with a wide range of infectious causes of IRIS. While the common cause of IRIS, regardless of the pathogen, may be due to restoration of an antigen-specific response, the immunopathogenesis of specific forms of IRIS, such as TB-IRIS, may be unique. Second, the diagnosis of IRIS is difficult. Although we used a strict definition for IRIS requiring the use of HIV RNA to objectively verify a positive response to ART, no gold standard definition or immune marker to define IRIS presently exists. Third, as with most immunological IRIS studies to date, ours was limited to the analysis of peripheral blood mononuclear cells with the assumption that the tissue-level immune response in IRIS is similar to that observed in the peripheral blood compartment. Future tissue-level IRIS immunology studies are needed, given others have demonstrated that non-IRIS HIV-infected patients on ART possess differences in immune activation patterns between tissue and peripheral blood [8]. Lastly, despite being one of the largest prospective immunological IRIS studies to date, the limited sample size and the low median CD4 cell count in our population may have limited our ability to examine immunological differences within T cells subsets, particularly within CD4^+ ^T lymphocyte subpopulations. Furthermore, this prospective study examined immunological difference between subjects after ART at the time of IRIS diagnosis and did not match subjects on baseline CD4 count. Thus, whether a difference in activation profiles exists between IRIS and non-IRIS subjects with similar baseline HIV RNA levels and CD4 cell counts remains to be determined.

In summary, in this prospective immunological study of IRIS in Sub-Saharan Africa, we observed heterogeneous T cell maturation among individuals experiencing IRIS and ART-matched controls and significant increases in activation markers in CD8^+ ^T cell effector subpopulations. These findings may therefore be useful in the development of future studies aimed at identifying patients at risk for the development of IRIS. Future prospective pathogen-specific IRIS immunological studies will likely require collaborative approaches and include measurement of effector CD8^+ ^and CD4^+ ^subpopulations, cytokine responses, and activation profiles in order to increase our understanding of its pathogenesis and identify potential biomarkers for the disease.

## Authors' contributions

DM and MS carried out the flow cytometry studies and drafted the manuscript. PM coordinated laboratory investigations. JO, DG, WS, and KW supervised laboratory efforts, provided technical laboratory expertise, and contributed to manuscript writing. DM, WDFV, AVR and CF conceived of the study and participated in its design and coordination and helped draft the manuscript. All authors read and approved the final manuscript.

## Conflict of interests

The authors declare that they have no competing interests.
